# Development, external validation and integration into clinical workflow of machine learning models to support pre‐operative assessment in the UK

**DOI:** 10.1111/anae.16777

**Published:** 2025-09-14

**Authors:** Alwyn Kotzé, Tom Lawton, Simon J. Howell, Ruairi O'Driscoll, Michael Odling‐Smee, Linqing Shangguan, Owen A. Johnson, David C. Wong

**Affiliations:** ^1^ Leeds Teaching Hospitals NHS Trust Leeds UK; ^2^ Faculty of Medicine and Health, University of Leeds Leeds UK; ^3^ Improvement Academy, Bradford Institute for Health Research Bradford UK; ^4^ Department of Computer Science University of York York UK; ^5^ Aire Logic Ltd Leeds UK; ^6^ School of Computing University of Leeds Leeds UK

**Keywords:** electronic health records, machine learning, pre‐operative care, risk assessment

## Abstract

**Introduction:**

Demand for surgical treatment is growing and patient complexity is increasing. The NHS England standard contract now requires that pre‐operative services risk stratify and optimise patients awaiting surgery. However, current pre‐operative workflows (whether electronic or paper‐based) remain based primarily on resource‐intensive manual tasks. Lack of real‐time data transfer has been identified as a key limitation to reducing the surgical backlog.

**Methods:**

We developed certified electronic linkages between a live pre‐operative assessment system (Smart PreOp, Aire Logic Ltd, Leeds, UK) and the GP Connect system from NHS England to retrieve clinical data directly from general practitioner records into pre‐operative questionnaires. We developed machine learning models to categorise patients into lower‐ and higher‐risk cohorts based on their predicted ASA physical status (1 or 2 vs. 3–5) and 30‐day postoperative mortality risk. In contrast with previous prediction modelling studies, we constrained variable selection from the outset to variables that are available electronically in real time for all UK surgical patients regardless of where they present (the proposed procedure, demographics and medications lists).

**Results:**

The development and external validation cohorts consisted of 110,732 and 67,878 patients, respectively, from two NHS Trusts using different electronic record systems. In external validation, at decision threshold 0.2, the ASA physical status prediction model had recall 0.69 and precision 0.95 for identifying lower‐risk (ASA physical status 1 or 2) patients. The mortality prediction model discriminated well in external validation but was poorly calibrated, lending support to the existing literature showing that hospital‐specific modelling improves mortality risk prediction. The technical architecture of the Smart PreOp system facilitates such hospital‐specific modelling and periodic model updates.

**Discussion:**

We conclude that conducting modelling together with systems development can yield accurate prediction models that may be implemented directly into electronic health records. A prospective study of clinical impact and acceptability is warranted.

## Plain Language Summary

More and more people need surgery, and many patients have health problems that make surgery riskier. NHS England now says that hospitals must check patients' health and make sure they are as well as possible before surgery. But the current way of doing this takes a lot of time and is mostly done by hand. One big problem is that information from doctors is not shared quickly, which slows things down and makes it harder to reduce the waiting lists for surgery. We built a safe computer link between a pre‐surgery system (called Smart PreOp) and the NHS GP Connect system. This means we can bring medical information straight from a patient's GP record into their pre‐surgery forms. We also created computer models (using machine learning) to sort patients into two groups: lower risk (fitter patients) and higher risk (patients with more health problems). The models used simple information that is always available for every surgery patient in the UK such as their planned operation; age; and list of medicines. We tested the models with information from nearly 180,000 patients across two different NHS hospitals. The model for spotting lower‐risk patients worked well: it found most of the right patients and made very few mistakes. The model for predicting who might die within 30 days after surgery was good at separating low and high risk but was not as accurate when looking at different hospitals. This shows that each hospital may need its own version of the model. The Smart PreOp system makes it possible to update these models for each hospital. We found that building the computer system and the prediction models together works best. This makes it easier to use the models directly in hospital systems. The next step is to test how well this works in real life and to see if patients and doctors find it helpful.

## Introduction

Demand for surgical treatment is growing, having outstripped capacity since before the COVID‐19 pandemic. As of November 2024, over 6 million patients in England were waiting for over 7 million procedures, with over 3 million patients missing the target set by NHS England for time to receiving first treatment [1]. From April 2024, the NHS standard contract mandates, for the first time, new responsibilities for provider hospitals such as early screening for modifiable risk factors, health optimisation and regular follow‐up while on the waiting list [2]. Workload for already stretched pre‐operative assessment teams can thus be expected to increase significantly [3], since triage is currently a manual process requiring substantial time and expertise [4, 5].

Improved use of data and digital systems has been identified as a key enabler of elective care recovery plans [3]. National reports and experts in the UK [6, 7, 8, 9] and elsewhere [10] recommend that care planning considers individualised objective risk assessment. However, the use of peri‐operative risk prediction models remains limited [10], despite a proliferation of new models being developed based on regression analysis [10] or machine learning [11]. A systematic review concluded that machine learning in peri‐operative medicine is still in an early stage of development [11]. Other than image analysis or precision oncology, artificial intelligence in healthcare remains confined to a few areas [12].

Several reasons have been identified for this lag, particularly in peri‐operative medicine. Issues of trust, bias and accountability are well described [13]. Despite rapid model proliferation, few models in peri‐operative medicine are externally validated and even fewer subjected to prospective study [11]. There is a lack of integration between models and clinical systems [14, 15]. In practice this means that, to quantify risk, a clinician must first open a website or app and transcribe data. Such additional manual work increases workload and potential for error, instead of making care safer.

Expertise for health data analytics and tools development to integrate prediction models with electronic health records is lacking in the NHS [16]. Currently available risk prediction models (e.g. surgical outcome risk tool (SORT) [17]) require information such as ASA physical status and some clinical assessment of risk, and others require laboratory test results [18, 19]. Modelling therefore cannot readily guide the assessment and testing process itself, despite national recommendations that patients expected to be straightforward are managed in dedicated pathways [2] or considered for elective surgery hubs and high‐volume, low‐complexity pathways [3].

We therefore aimed to develop a prediction model suite and accompanying technical infrastructure that can support pre‐operative assessment teams. This would automate data collection directly from the general practitioner (GP) record rather than relying on patient recollection and then feed that information into a prediction model to ensure that patients can be ‘streamed’ to appropriate pathways. To be suitable, the models must satisfy essential requirements such as: easy implementation in the NHS, by taking account only of data items that are available electronically and automatically for most surgical patients across the UK, regardless of where they live or where they are treated; external validation; integration with clinical workflow and appropriate certification; and inform care planning from receipt of referral onwards, including the pre‐operative assessment process itself.

## Methods

The NHS Personal Demographics Service [20] is the national master database of NHS patients, containing their NHS number and patient details. Data sharing networks between primary and secondary care are expanding rapidly [21]. Sharing is enabled and given a legal basis via the GP Connect systems [22]. Broadly, GP Connect allows approved healthcare workers to access a patient's GP record as an unstructured read‐only document (GP Connect HTML) or as a structured record that can be read and interpreted by software (GP Connect Access Record: Structured). As of February 2024, the only structured data items potentially available live were ‘medications’ and ‘allergies’. We therefore constrained our modelling from the outset to only use available items, namely demographics, medications and allergies, along with information the secondary care provider would always be expected to have, namely the proposed surgical procedure and method of admission (elective or otherwise).

We chose two outcomes – ASA physical status and 30‐day mortality – that can be expected to influence the pre‐operative assessment process and peri‐operative care planning. The ASA physical status was assigned by the anaesthetist attending on the day of surgery (that is, the score assigned after diagnoses, observations and test results became available, and a face‐to‐face assessment was conducted). We dichotomised the ASA physical status (1 or 2 vs. 3–5) to approximate low‐ and higher‐risk pathways as suggested by the Getting It Right First Time programme [4]. All‐cause mortality 30 days after surgery was chosen to allow comparison of this work with models published previously, and because mortality risk is suggested as a key element of peri‐operative care planning, including critical care allocation and shared decision‐making consultations [4, 7].

A research dataset was assembled, using prospectively recorded administrative and clinical data from Leeds Teaching Hospitals NHS Trust (LTHT) linked with mortality data from the Office for National Statistics. De‐identification was conducted programmatically as part of the extraction code, in such a manner that the research team never had access to patient identifiable data. De‐identification included application of the national data opt‐out [23] at source, followed by cryptographic hashing and date stamp obfuscation. The data access request was reviewed by a data access committee in accordance with established LTHT standard operating procedures before receiving Caldicott Guardian approval. As a condition of data access, age was provided in 5‐year bands as a privacy safeguard. Health Research Authority review confirmed that the resultant de‐identified data did not require research ethics committee review. Following dataset assembly, data were released in the context of a collaboration agreement, data sharing agreement and data sharing contract. The cohort comprised all patients who underwent surgery in LTHT between April 2018 and October 2022. Office for National Statistics linkage was conducted in June 2023 to give a clear period for the recording of deaths. Surgery was defined as any procedure conducted in an operating theatre, not including patients who received critical care rather than surgery in an operating theatre because of limited critical care capacity. Leeds Teaching Hospitals NHS Trust also runs pathways for emergency surgery in otherwise stable patients, who are discharged home for expedited pre‐operative assessment and planned non‐elective admission.

After model development, we entered into a similar data sharing agreement and data sharing contract with the Bradford Institute for Health Research, to access the Connected Bradford data platform for the purpose of external validation. The public engagement, ethics and governance of the platform is described elsewhere [24].

We estimated a minimum sample size requirement of 1783 patients, based on guidance from Riley et al. [25] (online Supporting Information Appendix S1). In practice, we used all retrospective data available to us. Each data entry, representing a surgical procedure, included the following variables: age; sex; Index of Multiple Deprivation decile; medications; admission method; and procedure. We did not study individuals aged < 20 y (given age banding conditions we were unable to define ‘adult’ as > 18 y). Pre‐processing, feature engineering, model training and fine‐tuning were all built into a custom pipeline, including a warning flag that can generate warnings to clinicians if the input data falls outside training data ranges. The pipeline was housed in a secure cloud environment and included capabilities for fitting models, pre‐processing raw data and predicting probabilities as well as schema validation, which ensured compatibility with the expected data format.

Medications were retrieved from the LTHT electronic health records; the medicines list on admission was entered by clinical pharmacists for each patient as part of medicines reconciliation. Medications and procedures were encoded in several ways. We mapped medications to count‐encoded values for British National Formulary codes and counted items in British National Formulary subchapters. We mapped procedures to Offices of Population Censuses and Surveys (version 4) chapters and subchapters. We further used a combination of domain‐specific risk categories, based on clinical knowledge and a biomedical language representation model embeddings using Bidirectional Encoder Representations from Transformers for Biomedical Text Mining [26]. Language representation models effectively treat a combination of text strings as ‘sentences’ by assigning a vector to each text string. A combination of similar vectors thus yields a similar endpoint in a theoretical n‐dimensional space, which maximises the information gain by capturing interactions. A numerical representation of each individual endpoint was used for modelling. All engineered features were used in model training. Patients without an ASA physical status recorded were not studied, as a target is necessary for supervised learning. Patients with missing predictors were included in the analysis (see below).

Model training followed the recommendations of the TRIPOD statement [27], since a machine learning‐specific version (TRIPOD‐ML) was still in development [28]. We used SciKit‐Learn in Python for model development (versions 1.3.2 through to 1.5.2). We developed models using Logistic Regression and XGBoost [29], a gradient boosting model that is well‐established for modelling tabular data [30]; XGBoost also provided a means of dealing with data sparsity (missing predictors) [29]. We split the LTHT data randomly (80:10:10) into training, testing and validation sets. The split was by patient ID to ensure that no data leakage could occur if the same patient appeared in training and validation sets for different procedures. We then conducted initial model training and iteration using the training and testing sets. To deal with class imbalance we optimised a set of hyperparameters that included the number of estimators (trees); max_depth; learning rate; and sample weighting. We computed the following measures of model performance against the LTHT validation set: area under the receiver operating characteristic (AUROC); precision; and recall. Calibration was assessed by means of calibration plots.

**Figure 1:**
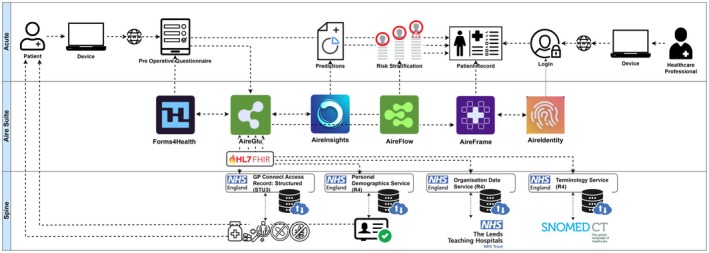
Smart PreOp system architecture and connections with NHS England digital services.

The final models were evaluated against the Connected Bradford cohort [24]. This validation included a de facto validation step of the embeddings process as medication recording formats differed between the two NHS Trusts. The Connected Bradford cohort contains medications as recorded in the patient's GP record. We used the GP medicines list 4 weeks before surgery for modelling to ensure maximal fidelity in clinical practice. We included adults aged > 18 y in external validation. We pre‐specified a sensitivity analysis to quantify the loss of performance if variables were missing at prediction. We also conducted post‐hoc sensitivity analyses. The models were integrated into the Aire Logic electronic pre‐operative assessment system, as well as via custom application programming interfaces with the GP Connect infrastructure. The application programming interfaces were evaluated by NHS Digital from technical, safety and Information Governance perspectives, including reference to the Medicines and Healthcare products Regulatory Agency [31]. Figure 1 illustrates the system architecture.

## Results

The LTHT cohort comprised 110,732 adult patients, who underwent 151,832 surgical procedures during 139,728 hospital admissions. The Connected Bradford cohort comprised 67,878 adult patients, who underwent 97,522 surgical procedures during 95,530 hospital episodes. Leeds Teaching Hospitals NHS Trust mandates recording of ASA physical status in the operating theatre management system as part of the World Health Organization surgical safety sign‐in checklist (Table 1). Only 2437 patients in the Connected Bradford cohort had ASA physical status recorded electronically. In the LTHT cohort, 71,924 (59.3%) patients had no medications recorded (online Supporting Information Table S1). This may be due to missingness or to patients not being on any medications; the training data did not differentiate. We treated all as ‘no medications recorded’. The embeddings process performed well. Across circa 9000 different medications in the LTHT and Connected Bradford cohorts, only one was matched by a conventional text‐matching strategy. However, all medicines were clustered successfully by the embeddings.

**Table 1:** Characteristics of the training/internal and external validation cohorts. Age, sex and deprivation index are given at time of first surgical procedure. Values are number (proportion).

	Training and internal validation set (LTHT)	External validation set (Connected Bradford)
**Age; y**		
< 20	0	750 (1.1%)
20–29	13,785 (12.4%)	7788 (11.5%)
30–39	17,060 (15.4%)	9568 (14.1%)
40–49	14,398 (13.0%)	8949 (13.2%)
50–59	18,420 (16.6%)	11,010 (16.2%)
60–69	19,153 (17.3%)	11,741 (17.3%)
70–79	18,005 (16.3%)	10,260 (15.1%)
≥ 80	9911 (9.0%)	7812 (11.5%)
Sex; male	51,415 (46.4%)	31,716 (46.7%)
**Index of Multiple Deprivation decile**		
1	25,978 (23.5%)	19,755 (29.1%)
2	11,755 (10.6%)	5661 (8.3%)
3	10,059 (9.1%)	7128 (10.5%)
4	5796 (5.2%)	4731 (7.0%)
5	9703 (8.8%)	4623 (6.8%)
6	9449 (8.5%)	4118 (6.1%)
7	11,684 (10.6%)	2637 (3.9%)
8	9481 (8.6%)	2352 (3.5%)
9	8810 (8.0%)	1723 (2.5%)
10	7825 (7.1%)	840 (1.2%)
Unknown	192 (0.2%)	14,310 (21.1%)
Total	110,732	67,878
**ASA physical status**		
1	31,594 (20.8%)	583 (23.9%)
2	79,523 (52.4%)	1404 (57.6%)
3	36,222 (23.9%)	429 (17.6%)
4	4230 (2.8%)	20 (0.8%)
5	263 (0.2%)	1 (<0.1%)
Total	151,832	2437
Elective surgery	105,794 (69.7%)	69,437 (71.2%)
30‐day mortality	2065 (1.4%)	1091 (1.1%)

LTHT, Leeds Teaching Hospital Trust.

**Figure 2:**
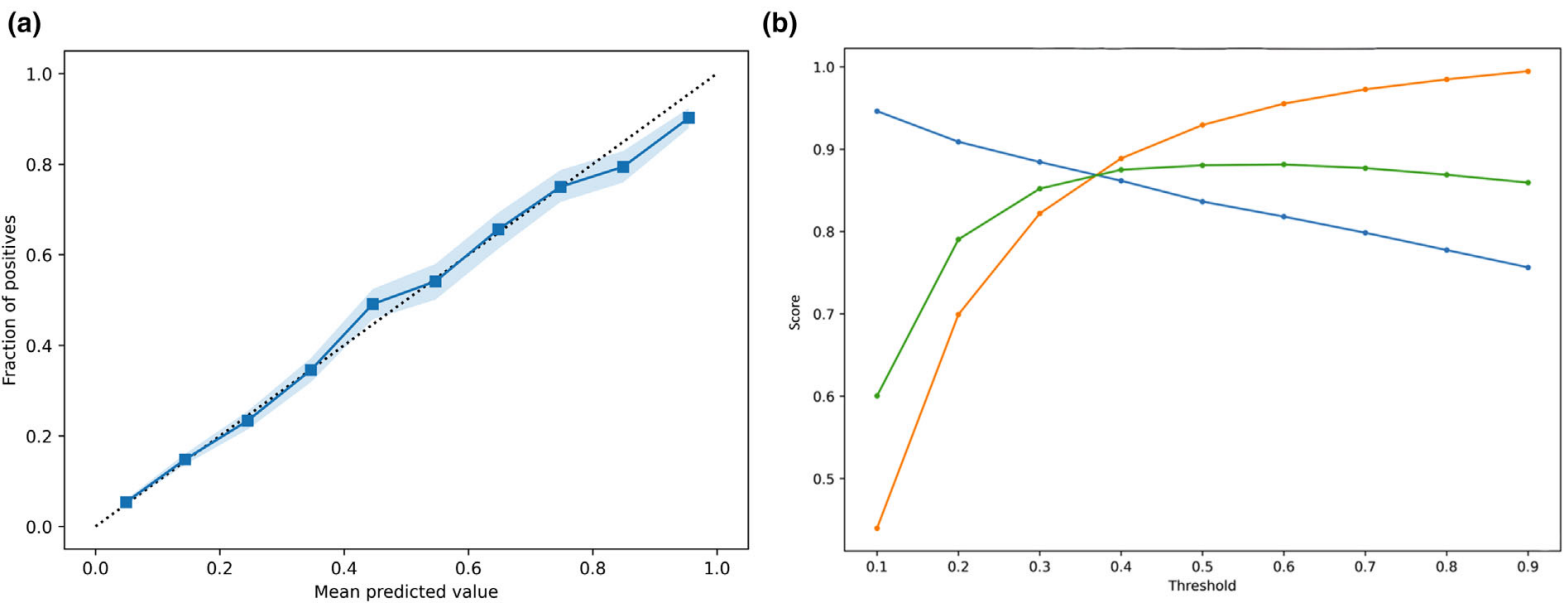
(a) Logistic regression model calibration plot and (b) precision/recall at different prediction thresholds, for prediction of ASA physical status 1–2 vs. 3–5, in the held‐out Leeds Teaching Hospitals NHS Trust internal validation set. Dotted line, perfect calibration; blue squares, model; blue shading, 95%CI; blue circles, precision; orange circles, recall; green circles, F1.

**Figure 3:**
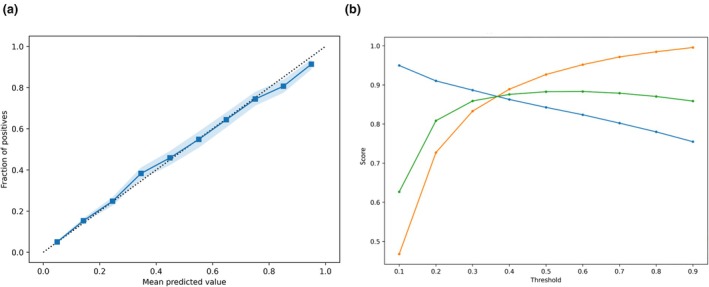
(a) XGBoost model calibration plot and (b) precision/recall at different prediction thresholds, for prediction of ASA physical status 1–2 vs. 3–5, in the held‐out Leeds Teaching Hospitals NHS Trust internal validation set. Dotted line, perfect calibration; blue squares, model; blue shading, 95%CI; blue circles, precision; orange circles, recall; green circles, F1.

**Figure 4:**
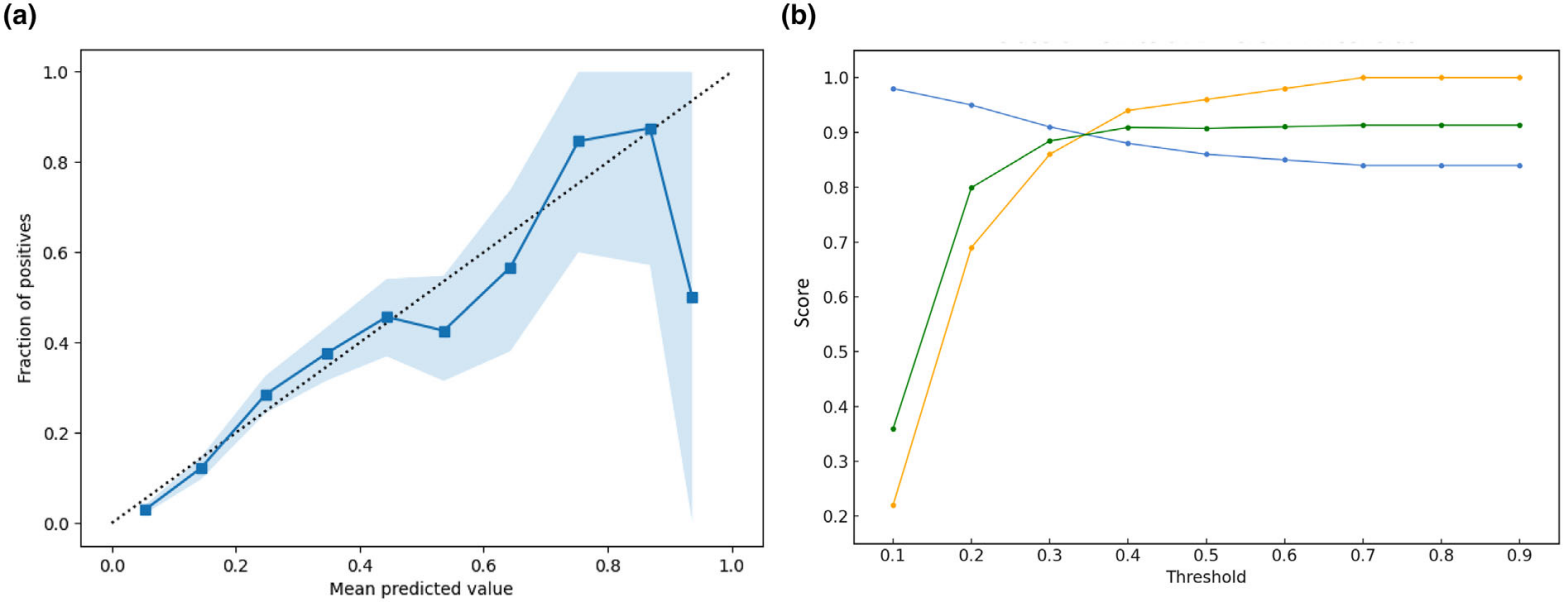
(a) External validation calibration plot; and (b) precision/recall at different prediction thresholds, for prediction of ASA physical status 1–2 vs. 3–5. Dotted line, perfect calibration; blue squares, model; blue shading, 95%CI; blue circles, precision; orange circles, recall; green circles, F1.

In the internal validation set, the logistic regression model for ASA physical status categorisation into 1–2 vs. 3–5, trained using all features, exhibited an AUROC of 0.81 (95%CI 0.80–0.82) and was well calibrated. At decision threshold 0.2, recall, precision and F1 score were 0.70, 0.91 and 0.79, respectively (Fig. 2). The comparable XGBoost model AUROC was 0.85 (95%CI 0.84–0.86) and was well calibrated. At decision threshold 0.2, recall, precision and F1 score were 0.73, 0.92 and 0.81, respectively (Fig. 3). Further external validation and sensitivity analyses were therefore conducted using XGBoost models only. In the external validation set, the XGBoost ASA physical status model AUROC was 0.81 (95%CI 0.77–0.92) and was well calibrated. At decision threshold 0.2, recall, precision and F1 score were 0.69, 0.95 and 0.80, respectively (Fig. 4).

The mortality prediction model (developed using XGBoost) AUROC was 0.86 (95%CI 0.83–0.88) and good calibration in the internal validation set. In the external validation set, it retained good discrimination (AUROC 0.87, 95%CI 0.84–0.91) but systematically overpredicted mortality. Further analyses on mortality prediction are in online Supporting Information Figures S1–S3.

Due to the small number of Connected Bradford procedures with recorded ASA physical status, we did not conduct sensitivity analyses in the external validation cohort. The XGBoost ASA physical status model retained discrimination (AUROC 0.81, 95%CI 0.78–0.89) and calibration if the surgical procedure was masked from the model at the prediction stage in the LTHT cohort (online Supporting Information Figure S4).

In post‐hoc analyses, we re‐trained the XGBoost model on a population that did not include non‐elective admissions, to simulate a setting that, unlike LTHT, does not conduct pre‐operative assessment for some urgent surgery. The AUROC was 0.83 (95%CI 0.82–0.84) with good calibration. At decision threshold 0.2, the recall, precision and F1 score were 0.67, 0.92 and 0.83, respectively (online Supporting Information Figure S5). We also conducted decision curve analysis to evaluate the clinical utility of the XGBoost model across a range of thresholds [31]. Compared with the default strategy of classifying all as low or all as high‐risk, the model yielded net benefit across all thresholds (online Supporting Information Figure S6).

We compared the mortality rates of correctly‐ and incorrectly‐classified ASA physical status 1–2 and 3–5 cohorts of the validation sets. This was a safety check to establish the risk profile of patients where the model assigned the patient to a different category than the ground truth (the anaesthetist). For patients who were ASA physical status 1–2 (ground truth), the mortality rate was significantly higher for the subset where the model assigned ASA physical status 3–5 (15/829, 1.8% vs. 18/10,340, 0.2%; p < 0.001). Conversely, where the model assigned ASA physical status 1–2 to true patients with ASA physical status 3–5, the mortality rate was lower (52/1933, 2.7% vs. 111/2192, 5.1%; p < 0.001) (online Supporting Information Figure S7).

The model prediction pipeline was integrated with the existing electronic pre‐operative assessment solution from Aire Logic Ltd., including functionality for recording patient consent for data sharing; electronic pre‐operative assessment questionnaires; GP Connect access; and clinic workflows. The system is highly customisable using a low‐code approach, enabling user organisations to configure many aspects of the system for local circumstances and pathways without requiring new code [32]. Examples of individual output and clinic workflows are displayed in online Supporting Information Figures S8 and S9.

## Discussion

We present externally validated models developed from a large cohort of surgical patients. In addition, we produced a pipeline for recording patients' medication lists automatically in an electronic pre‐operative assessment system directly from their GP record, rather than relying on patients self‐reporting their medication histories. This pipeline will also be suitable for importing other information (e.g. diagnoses, vital sign observations, test results) from GP records when this functionality is made available by NHS England. We also developed a well‐performing machine learning model for automated risk stratification that is aimed specifically at supporting provider organisations in implementing NHS England contractual requirements on early risk stratification [2].

In contrast with previous model developments, we ensured from the outset that the prediction variables and technical architecture were suitable for implementation within routine pathways and integrated with a live electronic pre‐operative assessment system as proof‐of‐concept. The technical pipeline performs well across different electronic patient record systems. The ASA physical status model can identify lower‐risk (ASA 1–2) patients with > 90% precision. The model differed from anaesthetists in the category it assigns in genuine edge cases, where mortality risk is intermediate between the rest of the ASA physical status 1–2 and 3–5 cohorts. The mortality model discriminated well in external validation but was poorly calibrated. The Smart PreOp system is first‐of‐type, combining real‐time structured data transfer from primary to secondary care with machine learning augmentation.

Despite rising demand [1] and complexity [33], surgical pathways are still based largely on manual processes. Digital questionnaires are likely to increase efficiency somewhat [34] but remain manual processes substituting paper for devices. Triage by means of screening tools and read‐only GP records access is a time‐consuming task requiring expertise [4]. Furthermore, the fidelity of suggested stratification checklists [4] has not been evaluated formally, though they have face validity. ‘Radical redesign’ of surgical pathways to improve outcomes has been advocated for some time [35] and is congruent with recent guidelines [34] and NHS contractual mechanisms [2]. However, there is a risk that incremental changes (e.g. from paper questionnaires to the same questionnaires via digital means or the addition of screening steps to current pathways) will be insufficient. The potential for shared decision‐making and health optimisation will inevitably be diluted if pre‐operative teams are primarily focused on keeping manual processes going.

Industry machine learning models (e.g. in financial services) typically arise directly from specific business questions [36, 37] and are integrated into systems from the outset. Conversely, machine learning in peri‐operative medicine is primarily an academic activity focused on modelling per se, with translation to routine practice often not being a focus [11]. Incorporating models into software is thus left to electronic health record providers or NHS Trusts. The Goldacre review identified the lack of widespread advanced analytical expertise in the NHS as a shortcoming in the system [16]. In practice, software for implementation at scale can thus only be developed in collaboration with industry, at least until data analysis and software engineering is professionalised in the NHS or a funding system is created for open source clinical tools development [16]. However, large USA‐based electronic health record vendors have implemented paid‐for proprietary models without external validation, which were subsequently shown to perform poorly [38].

We aimed to show that a model developed in response to a specific NHS business question, in this case the requirement to stratify patients efficiently, so releasing pre‐operative teams to focus on optimisation for those that are likely to need it. This model can be developed from data that can be retrieved automatically, again releasing pre‐operative staff time. We balanced discrimination against precision and recall, since correctly ranking patients in order is arguably less relevant to clinical decision‐making than whether predicted risk is accurate at the decision threshold [39, 40]. Crucially, we wanted to release validation data and develop a technical pipeline for periodic model updating. The performance of even well‐validated models can deteriorate over time as populations and clinical practice change, making evaluation a continuing task [41].

Given that only medicines, allergies and demographics are currently available for structured transfer, the bar for model performance was high. The ASA physical status machine learning model had to predict, from a few data items, the ASA physical status score category (1–2 vs. 3–5) anaesthetists would likely assign after a comprehensive pre‐operative assessment, with all the subjective information that such a consultation generates. Inter‐rater variability in ASA physical status scoring is a well‐described issue, with a third of patients assigned different scores in a pre‐operative assessment clinic compared with on the day of surgery [42], and at best fair agreement being found between anaesthetists faced with the same information [43, 44]. When setting a prediction target of the ASA physical status score itself (from 1 to 5) the concept of a firm ‘ground truth’ thus does not exist; had the same patient been scored by a different anaesthetist, the score may well have been different. However, most scores were within one grade of each other [42].

In practice, clinical pathways are often dichotomised with lower‐risk patients and procedures being considered for high‐volume, low‐complexity pathways [45] and objective assessment of mortality risk to guide decision‐making being recommended for patients with ASA physical status 3–5 [46]. A categorisation model (ASA physical status 1–2 vs. 3–5) is thus congruent with the business question, namely stratification rather than ASA physical status scoring per se. On this background, we achieved a well calibrated and discriminating model. In our external validation cohort, the missingness of ASA physical status scores cannot be assumed to be at random. Also, the medicines were retrieved from primary care records, not secondary care as in the training and test sets. It thus likely constitutes a truly independent cohort, suggesting that the ASA physical status model will perform well across diverse settings.

We also evaluated ASA physical status model failure, comparing correctly‐ and incorrectly‐classified patient episodes with the assessment by anaesthetists of whether the patient is likely to be of low (physical status 1–2) or higher (physical status 3–5) complexity. Patient outcomes were more congruent with model categories than with individual assessments. This may again be explained by the modelling process where a model, in effect, learns what the most common or likely category is for a given combination of data and will assign that consistently. The implication is therefore not that a model can somehow be better than an anaesthetist at stratification, rather that it can eliminate inconsistency. In this way it approximates ‘anaesthetic consensus’, congruent with research into inter‐rater variability which defined the notion of a ‘correct’ ASA physical status by consensus [43].

Our mortality prediction model was well calibrated and had discrimination statistics in a similar range to SORT, which displayed the best combination of usability and performance in a recent systematic review [10]. It retained discrimination in external validation but systematically over‐predicted risk. This finding adds weight to a recent model development study by Oliver et al., who found that hospital‐specific modelling was necessary for optimal performance [19]. Machine learning models using between 31 and 285 input variables [11] that display similar discrimination performance are not currently suitable for implementation at scale in the UK without electronic patient record integration, for which NHS expertise is scarce [16].

A logistic regression model using the same engineered features as the XGBoost model exhibited a clinically similar performance. This is in keeping with a systematic review which found no benefit of using machine learning over regression techniques [47]. However, the technical and access requirements for using GP Connect to automate collection of the medicines list are identical, regardless of which modelling is performed subsequently. In view of the incremental but statistically significant improvement using XGBoost in this case, we opted to conduct further evaluation using XGBoost. In practice, a deploying organisation can configure the system to use either model.

A key aim of the project was to address the ‘research‐practice’ gap and integrate any model result into digital workflows suitable for implementation into clinical practice [48]. Producing a model alone, without providing practical means to use it and completing the regulatory steps necessary for implementation, would add to the gap rather than address it. The fact that the outputs are probabilities, rather than classifications, enables a deploying organisation to choose a prediction threshold appropriate for its clinical context. For example, a case for assigning lower‐risk patients to expedited pathways may have a low‐risk tolerance and thus choose a high precision model threshold. This is in keeping with medicolegal guidance that clinical artificial intelligence systems should output probabilities rather than make recommendations, at least until the regulatory system evolves to match technical developments [49]. Performance was also maintained if the surgical procedure was unknown, which implies potential use cases for risk stratification at referral from primary care (rather than on receipt of referral in secondary care), enabling better use of high‐volume, low‐complexity surgical hubs. The computing and time requirements for modelling are such that real‐time use is feasible and that predictions may be repeated regularly while a patient is on the waiting list to continually monitor risk and flag any changes beyond preset thresholds to clinicians. The advantage of our model over SORT is that neither an ASA physical status score nor clinical judgement on risk is required; the models rely solely on data already in the system at the point of decision to offer surgery. Even without modelling, automating data collection from GP records may reduce the burden of questionnaire completion for patients, as well as being less susceptible to errors related to manual data entry and/or transcription between systems.

A further strength of the project was developing the technical and regulatory infrastructure [16]. Our methods and model development systems can be turned rapidly to modelling for other outcomes, or for regular updates and hospital‐specific model development as recommended by others active in this field [19]. The relatively limited number of variables currently available was a limitation of this study. However, the GP Connect development roadmap [22] is soon to increase the range of variables available for structured data transfer. When diagnoses, observations and laboratory tests become available via GP Connect, we plan to develop new models that incorporate these data points. The availability of more structured data will also likely reduce the burden on patients, as patient‐completed forms could become shorter and less onerous.

Our work has limitations. A high proportion of our training data had no medications recorded which may be either due to missingness or to the patient being on no medications, and the training data did not differentiate. We treated all as ‘no medicines’. In future iterations, when missingness and no medications can be distinguished, we would expect model performance to improve. Since feature engineering was complex, involving both custom classifications and embeddings, the model outputs are not interpretable or explainable to the same extent as currently recommended regression‐based prediction models (e.g. SORT). It is important to note that this is a consequence of much of feature engineering as the prediction modelling itself; as such, this also applies to our logistic regression model. This concern is mitigated somewhat by the outputs being probabilities, as discussed above. Whether our models are equitable and whether their predictions differ significantly based on protected characteristics such as sex or ethnicity also requires specific evaluation. Safety, acceptability and clinical impact (or lack thereof) in live use will require prospective study.

In conclusion, we developed the first‐of‐type electronic pre‐operative assessment system that incorporates real‐time data transfer between primary and secondary care, combined with machine learning augmentation to address a key healthcare business requirement. The automated machine learning models perform at least as well as manual triage at initial stratification, with precision of around 95% in identifying low‐risk patients. This work acts as proof‐of‐concept showing that model development can be integrated with NHS systems from the outset rather than post‐hoc, producing accurate models without compromising clinical utility.

## Supporting information


**Plain Language Summary**.

**Appendix S1.** Sample size calculation.

**Table S1.** Missingness summary.

**Figure S1.** 30‐day postoperative mortality model performance in internal validation.
**Figure S2.** 30‐day postoperative mortality model performance in external validation.
**Figure S3.** 30‐day postoperative mortality model performance, model fine‐tuned using Connected Bradford dataset.
**Figure S4.** Sensitivity analysis with procedure code masked from model.
**Figure S5.** Model retrained on elective surgery population.
**Figure S6.** Decision curve analysis.
**Figure S7.** 30‐day postoperative mortality for correctly and incorrectly‐classified groups.
**Figure S8.** Example individual output generated from hypothetical data.
**Figure S9.** Example clinical workflow generated from hypothetical records.
