# Supplementary material for: Development, external validation and integration into clinical workflow of machine learning models to support pre‐operative assessment in the UK
Source: Anaesthesia. 2025 Sep 14;81(2):201–12. doi: 10.1111/anae.16777 (PMC12803613; doi:10.1111/anae.16777)
Supplement: Supplementary file 1 — Plain Language Summary. [file ANAE-81-201-s004.docx]

**Plain Language Summary**

More and more people need surgery, and many patients have health problems that make surgery riskier. NHS England now says that hospitals must check and prepare patients properly before surgery. But the current way of doing this takes a lot of time and is mostly done by hand. One big problem is that information from doctors is not shared quickly, which slows things down and makes it harder to reduce the waiting lists for surgery. We built a safe computer link between a pre-surgery system (called Smart PreOp) and the NHS GP Connect system. This means we can bring medical information straight from a patient’s GP record into their pre-surgery forms. We also created computer models (using machine learning) to sort patients into two groups: lower risk (fitter patients); and higher risk (patients with more health problems) The models used simple information that is always available for every surgery patient in the UK such as their planned operation; age; sex; and list of medicines. We tested the models with information from nearly 180,000 patients across two different hospitals. The model for spotting lower-risk patients worked well: it found most of the right patients and made very few mistakes. The model for predicting who might die within 30 days after surgery was good at separating low and high risk but was not as accurate when looking at different hospitals. This shows that each hospital may need its own version of the model. The Smart PreOp system makes it possible to update these models for each hospital. We found that building the computer system and the prediction models together works best. This makes it easier to use the models directly in hospital systems. The next step is to test how well this works in real life and to see if patients and doctors find it helpful.
