# Supplementary material for: Development, external validation and integration into clinical workflow of machine learning models to support pre‐operative assessment in the UK
Source: Anaesthesia. 2025 Sep 14;81(2):201–12. doi: 10.1111/anae.16777 (PMC12803613; doi:10.1111/anae.16777)
Supplement: Supplementary file 2 — Appendix S1. Sample size calculation. [file ANAE-81-201-s003.docx]

**Appendix S1 Sample size calculation**

**Key facts**

- Intention is to create a binary prediction model to predict either ASA score (dichotomized) or 30-day mortality
- Number of candidate predictors: 5 [Age, sex, IMD, admission method, procedure] + 16 related to medication (15 counts of BNF chapter medication, 1 summary of BioBERT embedding) = 21 predictors
- case mix: ASA low/high ratio = 70/30. Mortality dead/live ratio = 1/99

Minimum sample size is based on recommendations from Riley et al. *Calculating the sample size required for developing a clinical prediction model*

**Estimate overall outcome proportion with sufficient precision**

ASA:

Mortality:
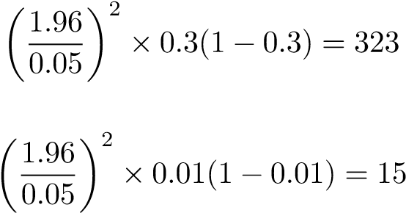


**Target a small mean absolute prediction error**


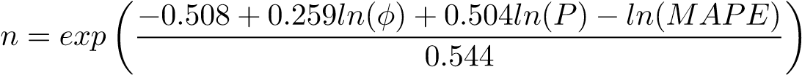


setting MAPE = 0.050, in a setting with anticipated outcome proportion of 0.3 (for ASA) and 0.01 (for mortality) and 21 candidate predictor parameters, we require: ASA (*ϕ* = 0*.*3*,P* = 21*,MAPE* = 0*.*05):


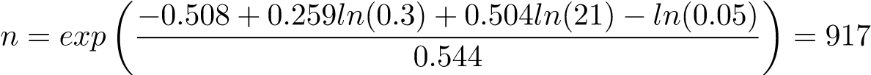


mortality (*ϕ* = 0*.*3*,P* = 21*,MAPE* = 0*.*05):


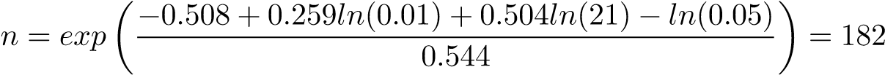


1

**Target a shrinkage factor of 0.1**

Assuming of at least
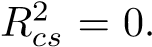
1 (more conservative than the recommendation in the absence of other prior knowledge from Riley et al.)


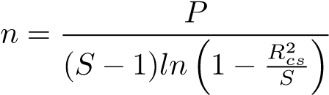


to target an expected shrinkage of 0.9, we need:


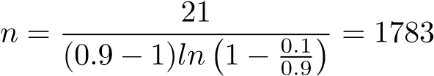


**Target small optimism of 0.05 in the apparent** *R*^2^

Calculate shrinkage factor that corresponds to an expected optimism of *δ*:


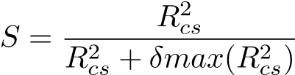


We use *R_cs_*^2^ = 0*.*1 as before. *max*(*R_cs_*^2^ ) is calculated as:


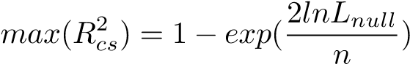


where:


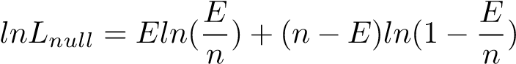


n is an arbitrary chosen sample size, and E is the total number of people with the outcome present. For ASA, in which *E* = 300 when *n* = 1000:


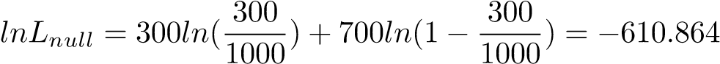


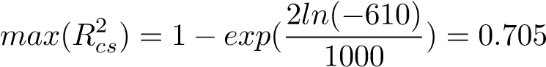


so S and n are estimated as:


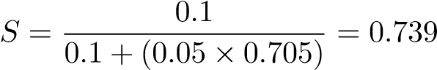


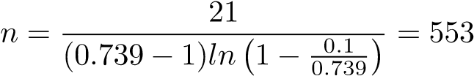


Repeating this the mortality model in which *E* = 10 when *n* = 1000 will result in a smaller n.

**Summary**

The minimum sample size required is the maximum of the four criteria: *n* = 1783, far less than the actual training cohort size of 110,732
