# Supplementary material for: Development, external validation and integration into clinical workflow of machine learning models to support pre‐operative assessment in the UK
Source: Anaesthesia. 2025 Sep 14;81(2):201–12. doi: 10.1111/anae.16777 (PMC12803613; doi:10.1111/anae.16777)
Supplement: Supplementary file 3 — Table S1. Missingness summary. [file ANAE-81-201-s001.docx]

# **Table S1.** Missingness summary. The percentage of missing data for each category is presented in the Table below.

| **Variable** | **Missingness percentage (N)** |
| --- | --- |
| Age | 0 (0) |
| Sex | 0 (0) |
| IMD Decile | 0.15 (183) |
| Medications | 59.29 (71924) |
| Admission Method | 3.10 (3766) |
| Procedure | 7.20 (8737) |

We note that missingness in *Medications* may be due to either absence of medication use, or missing data. In the absence of other information, we treated both mechanisms equally, replacing the missing variable with ‘no medications’.
