# Supplementary material for: Development, external validation and integration into clinical workflow of machine learning models to support pre‐operative assessment in the UK
Source: Anaesthesia. 2025 Sep 14;81(2):201–12. doi: 10.1111/anae.16777 (PMC12803613; doi:10.1111/anae.16777)

# **Fig. S1**. 30-day postoperative mortality model performance in internal validation


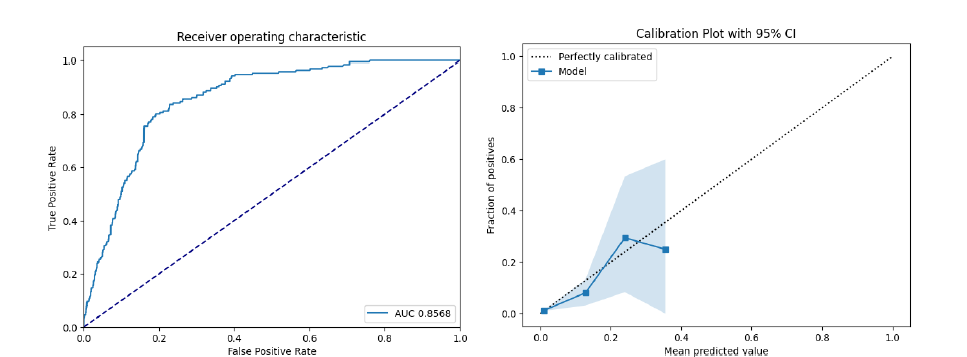


# **Fig. S2.** 30-day postoperative mortality model performance in external validation


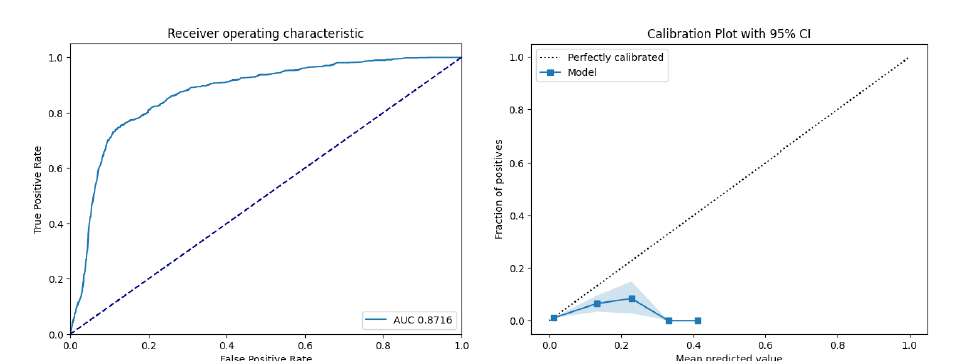


# **Fig. S3.** 30-day postoperative mortality model performance, model fine-tuned using Connected Bradford dataset


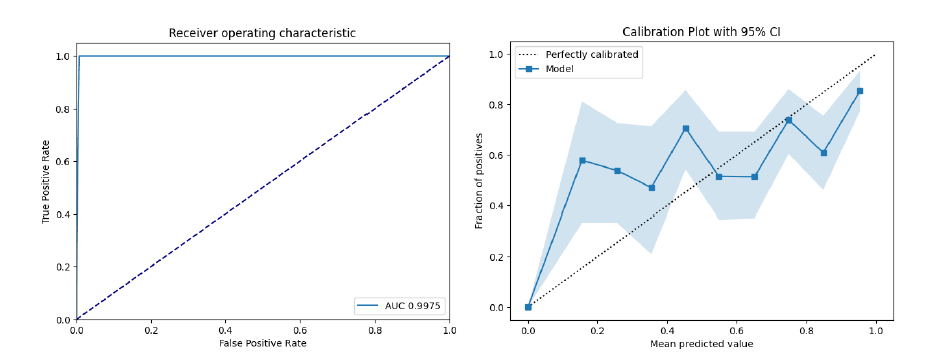


# **Fig. S4.** Sensitivity analysis with procedure code masked from model


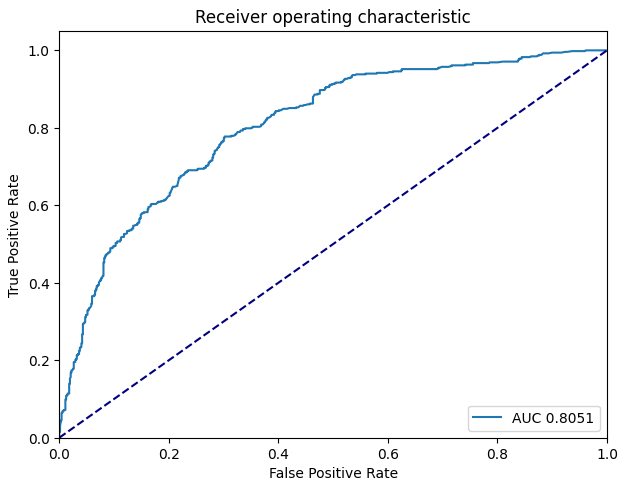

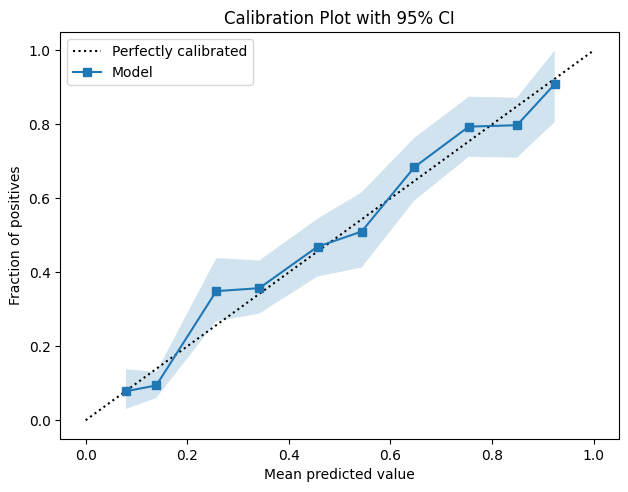


# **Fig. S5.** Model retrained on elective population. We retrained the XGBoost model including only the elective population (excluding admission methods with codes: [21, 24, 2B, 2D]. The elective population dataset contained 71% of the original dataset. Model AUC = 0.8311


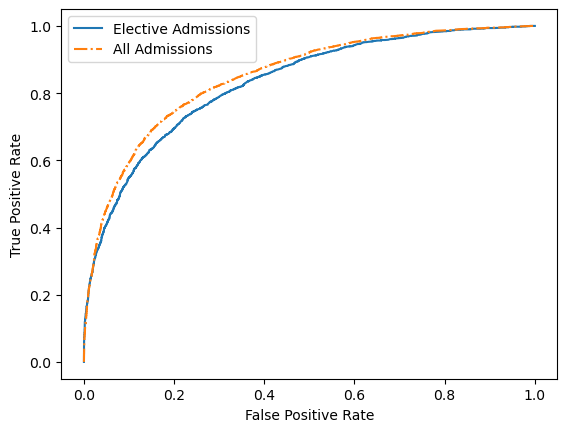


# **Fig. S6**. Decision Curve Analysis. A decision curve analysis plot, using internal validation results on the XGBoost model trained on all data to predict dichotomised ASA categories. Assuming a well-calibrated model, and that we would wish to assign roughly a 1:4 proportion to the high risk group (to ensure that there is no additional economic cost), then a decision threshold of 0.5 is plausible. At this operating point, and indeed, over all points, the model confers benefit over assigning all to the low ASA group or assigning all to the high ASA group.


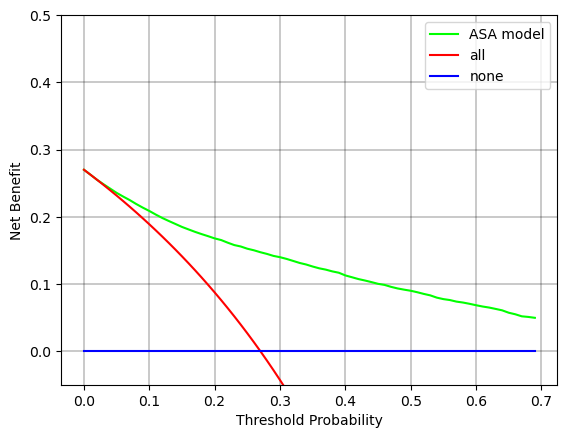


# **Fig. S7.** 30-day postoperative mortality for correctly- and incorrectly-classified groups


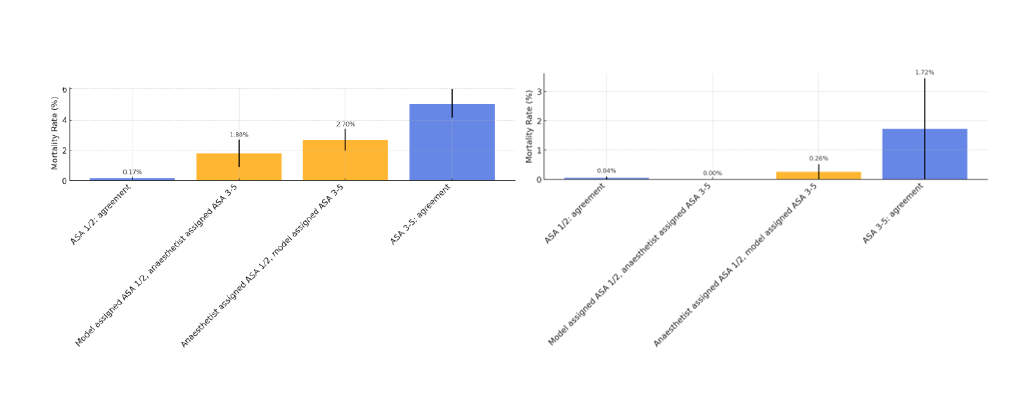


# **Fig. S8.** Example individual output generated from hypothetical data


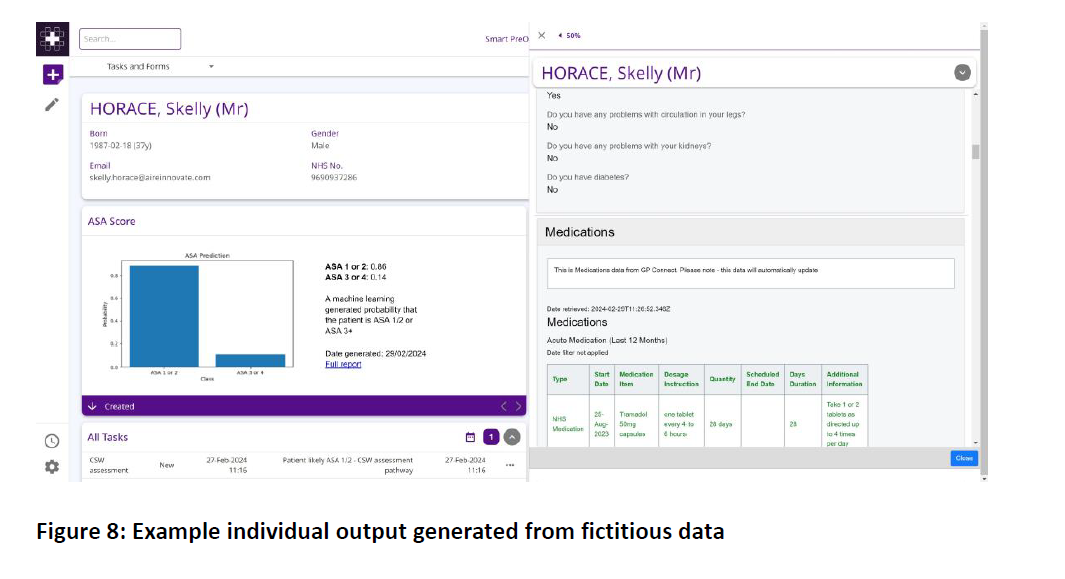


# **Fig. S9.** Example clinic workflow generated from hypothetical data


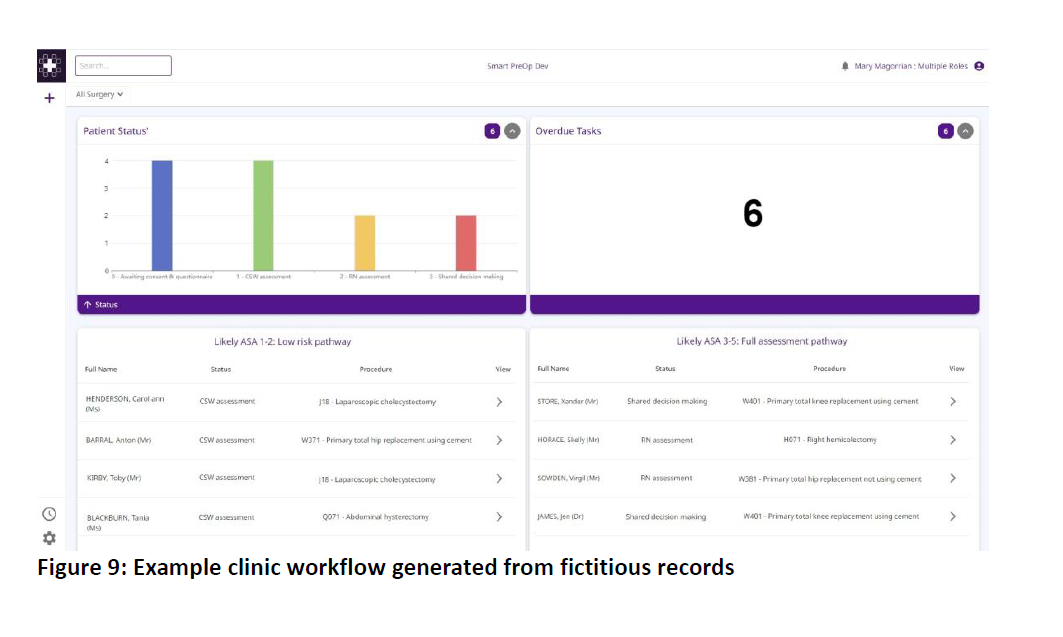

Supplement: Supplementary file 4 — Figure S1. 30‐day postoperative mortality model performance in internal validation. Figure S2. 30‐day postoperative mortality model performance in external validation. Figure S3. 30‐day postoperative mortality model performance, model fine‐tuned using Connected Bradford dataset. Figure S4. Sensitivity analysis with procedure code masked from model. Figure S5. Model retrained on elective surgery population. Figure S6. Decision curve analysis. Figure S7. 30‐day postoperative mortality for correctly and incorrectly‐classified groups. Figure S8. Example individual output generated from hypothetical data. Figure S9. Example clinical workflow generated from hypothetical records. [file ANAE-81-201-s002.docx]
